# Dynamic 3D Measurement Based on Camera-Pixel Mismatch Correction and Hilbert Transform

**DOI:** 10.3390/s25030924

**Published:** 2025-02-03

**Authors:** Xingfan Chen, Qican Zhang, Yajun Wang

**Affiliations:** Department of Opto-Electronics, Sichuan University, Chengdu 610065, China; 2022222050014@stu.scu.edu.cn (X.C.); zqc@scu.edu.cn (Q.Z.)

**Keywords:** 3D measurement, dynamic measurement, pixel mismatch correction, Hilbert transform

## Abstract

In three-dimensional (3D) measurement, the motion of objects inevitably introduces errors, posing significant challenges to high-precision 3D reconstruction. Most existing algorithms for compensating motion-induced phase errors are tailored for object motion along the camera’s principal axis (Z direction), limiting their applicability in real-world scenarios where objects often experience complex combined motions in the X/Y and Z directions. To address these challenges, we propose a universal motion error compensation algorithm that effectively corrects both pixel mismatch and phase-shift errors, ensuring accurate 3D measurements under dynamic conditions. The method involves two key steps: first, pixel mismatch errors in the camera subsystem are corrected using adjacent coarse 3D point cloud data, aligning the captured data with the actual spatial geometry. Subsequently, motion-induced phase errors, observed as sinusoidal waveforms with a frequency twice that of the projection fringe pattern, are eliminated by applying the Hilbert transform to shift the fringes by π/2. Unlike conventional approaches that address these errors separately, our method provides a systematic solution by simultaneously compensating for camera-pixel mismatch and phase-shift errors within the 3D coordinate space. This integrated approach enhances the reliability and precision of 3D reconstruction, particularly in scenarios with dynamic and multidirectional object motions. The algorithm has been experimentally validated, demonstrating its robustness and broad applicability in fields such as industrial inspection, biomedical imaging, and real-time robotics. By addressing longstanding challenges in dynamic 3D measurement, our method represents a significant advancement in achieving high-accuracy reconstructions under complex motion environments.

## 1. Introduction

Non-contact 3D shape measurement is vital in fields like industrial inspection, robotics, and virtual reality [1,2,3,4,5]. Among the many available techniques, phase-shifting profilometry (PSP) has gained significant popularity due to its high accuracy, fine resolution, speed, and robustness against noise. PSP works by capturing a series of fringe patterns at different phase shifts and reconstructing the 3D geometry of objects. However, a fundamental limitation of PSP lies in its assumption that both the object and the measurement system remain stationary during fringe acquisition. Any motion during this process introduces errors, particularly motion-induced phase errors, which can severely affect the accuracy of the 3D reconstruction [6,7].

To address these motion-induced errors, researchers have primarily explored two main approaches: hardware-based methods and algorithm-based methods. Hardware-based methods aim to minimize errors by using advanced, high-speed components to accelerate fringe projection and acquisition. While these methods are effective, they come with significant drawbacks, such as increased system costs and potential loss of spatial resolution [8,9,10,11,12,13,14,15]. Additionally, hardware improvements are not a fundamental solution, as errors can still occur if the motion exceeds the enhanced system speed.

Algorithm-based methods, on the other hand, focus on compensating for motion-induced errors without relying on expensive hardware. For instance, Guo et al. introduced Fourier-assisted methods utilizing dual-frequency gratings and Fourier fringe analysis to mitigate errors [16]. Similarly, Wang et al. proposed an iterative motion error compensation algorithm based on binary defocusing techniques. This approach estimates phase shifts using additional fringe pattern samples, but it has limitations due to its reliance on uniform motion assumptions and its neglect of camera-pixel mismatch caused by object motion [17]. Lu and Duan et al. developed methods involving marker tracking and reference planes to correct motion-induced errors. However, these methods are restricted to 2D motion scenarios and fail in cases of 3D random motion [18,19].

**Figure 1:**
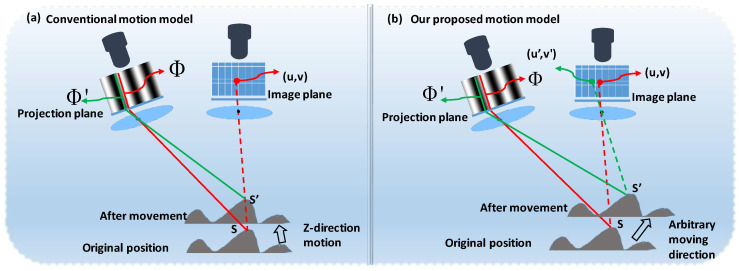
Different motion models. (**a**) Conventional model with nearly Z-direction motion assumption; (**b**) proposed model with arbitrary moving direction.

Most existing motion error compensation algorithms operate under a conventional assumption model, as shown in Figure 1a, which is only suitable for object motion along the camera’s principal axis (Z direction). However, real-world applications often involve combined motions in both the X/Y and Z directions, as depicted in Figure 1b. In such scenarios, motion-induced errors are more complex, consisting of both camera-pixel mismatch and phase-shift errors. Current compensation algorithms generally lack the capability to address these two error types simultaneously, leaving a significant gap in achieving accurate 3D reconstruction under complex dynamic conditions.

Our analysis reveals a strong correlation between camera-pixel mismatch and phase-shift errors, suggesting the potential for simultaneous compensation in 3D coordinate space. Based on this insight, we propose a universal motion error compensation algorithm that effectively addresses both types of errors, ensuring improved accuracy in 3D measurements. The proposed method begins by correcting pixel mismatch errors in the camera subsystem using adjacent coarse 3D point cloud data. This step ensures better alignment of captured data with the object’s actual geometry. After pixel mismatches are corrected, motion-induced phase errors, which manifest as ideal sinusoidal waveforms with frequencies twice that of the projection fringe pattern, are addressed. To eliminate these periodic phase errors, the Hilbert transform is applied to shift the fringes by π/2. These steps are iteratively refined until convergence is achieved, further enhancing the robustness of the compensation process.

Unlike conventional algorithms, our approach overcomes the strict motion assumption model and offers a systematic solution that compensates for both camera-pixel mismatch and phase-shift errors in 3D coordinate space. This method is particularly advantageous in scenarios involving complex, multidirectional motion, such as dynamic industrial inspection or real-time robotics applications. Experimental results validate the efficacy of the proposed algorithm, demonstrating its capability to achieve high-accuracy 3D reconstructions, even under challenging motion conditions. This advancement represents a significant step forward in the field of non-contact 3D shape measurement, offering broader applicability and practical solutions to longstanding challenges.

## 2. Principle

**Figure 2:**
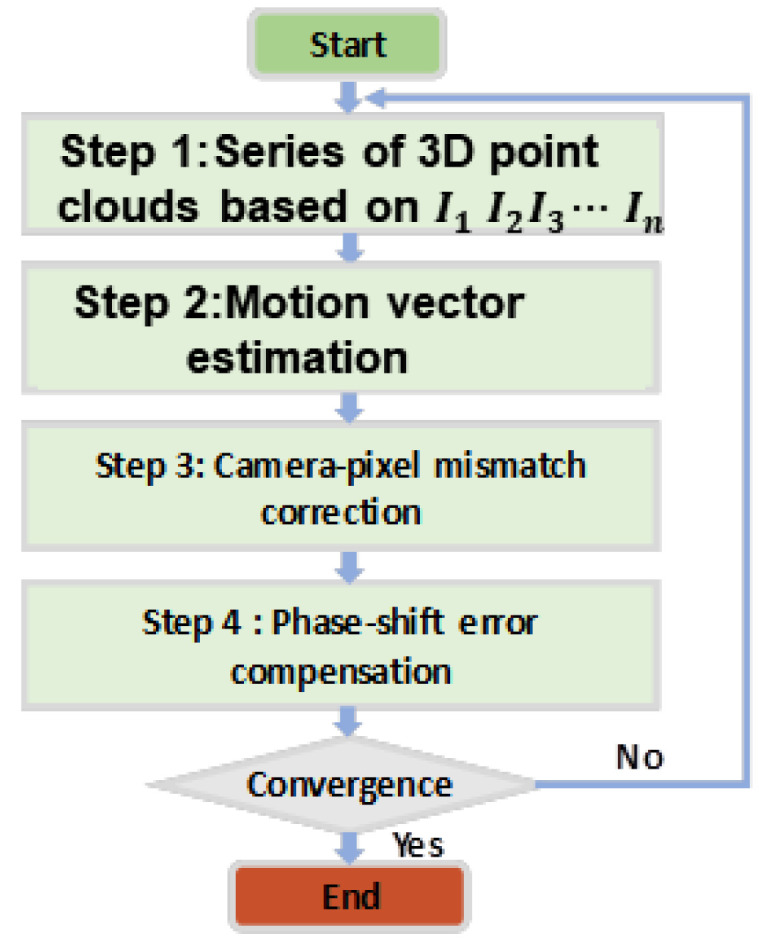
Overall flowchart of our proposed method.

The proposed general motion compensation strategy, as illustrated in Figure 2, involves a systematic process consisting of several key steps aimed at addressing motion-induced errors and ensuring high-accuracy 3D measurements. The major steps are as follows:

1. Acquisition of Rough 3D Point Clouds: The process begins with the use of the three-step phase-shifting method to acquire an initial set of rough 3D point clouds. This step serves as the foundation for further calculations and provides the spatial data needed to analyze motion-induced discrepancies.

2. Estimation of Motion Vectors: Next, motion vectors are estimated using a 3D centroid algorithm. This algorithm calculates the relative motion of the object by identifying and analyzing the changes in the spatial distribution of the 3D point clouds over successive frames. The estimated motion vectors play a critical role in understanding and correcting the displacement caused by object motion.

3. Correction of Camera-Pixel Mismatches: Using the motion vectors derived in the previous step, the algorithm proceeds to correct camera-pixel mismatches. This correction aligns the captured pixel data with the actual spatial position of the object, thereby addressing one of the primary sources of motion-induced errors. This step ensures that the data accurately reflect the object’s geometry, even under dynamic conditions.

4. Mitigation of Phase-Shift Errors Using the Hilbert Transform: After correcting camera-pixel mismatches, the next step involves applying the Hilbert transform to address phase-shift errors. The Hilbert transform shifts the fringes by π/2, effectively eliminating periodic phase errors that can arise due to motion. This step is crucial for refining the accuracy of the 3D reconstruction.

Overall, this strategy provides a comprehensive and integrated approach to motion error compensation. By systematically addressing both camera-pixel mismatches and phase-shift errors through iterative refinement, the proposed method enhances the robustness and precision of 3D measurements, making it suitable for applications involving complex dynamic conditions.

### 2.1. Rough 3D Reconstruction Using Phase-Shifting Algorithm

In the initial stage of the compensation algorithm process, we first need to use the traditional phase-shift algorithm to obtain rough 3D points. This allows us to estimate the motion vectors to the object’s centroid in the subsequent algorithm process. Assume that *k-*th fringe images of a generic *N*-step phase-shifting algorithm can be described as follows:

(1) $$I_{n}^{c}(x,y)=A^{c}(x,y)+B^{c}(x,y)\cos(\phi (x,y)+2\pi n/N)$$

Using the fringe patterns captured by the camera and applying phase-shifting and unwrapping algorithms, the absolute phase of the object is obtained. Then, by combining the camera and projector parameter matrices, the three-dimensional point cloud data can be calculated. The specific calculations are as follows:

(2) $$\left\{\begin{array}{l} u_{p}=\phi \times P/(2\pi )\\ s_{c}[\begin{array}{ccc} u_{c} & v_{c} & 1]=M_{c}[\begin{array}{cccc} X_{w} & Y_{w} & Z_{w} & 1] \end{array} \end{array}\\ s_{p}[\begin{array}{ccc} u_{p} & v_{p} & 1]=M_{p}[\begin{array}{cccc} X_{w} & Y_{w} & Z_{w} & 1] \end{array} \end{array} \end{array}\right.$$

where *P* represents the fringe period, and ϕ represents the absolute phase obtained through the phase-shifting algorithm. $u_{p}$, $v_{p}$ represent the horizontal and vertical coordinates of the projector, and similarly, $u_{c}$, $v_{c}$ represent the horizontal and vertical coordinates of the camera. *M_c_* and *M_p_* are the projection matrices of the camera and the projector.

The ϕ specific calculation process involves first using the phase-shifting method to obtain the wrapped phase.

(3) $$\phi (x,y)=\tan^{-1}\frac{\sum_{n=0}^{N-1}I_{n}^{c}(x,y)\sin(2\pi n/N)}{\sum_{n=0}^{N-1}I_{n}^{c}(x,y)\cos(2\pi n/N)}$$

where the value of ac tan is within [−π,π), thus ϕ(x,y) is not continuous. By applying a phase unwrapping algorithm to calculate the fringe order, the continuous phase ϕ can be obtained.

(4) ϕ=ϕ(x,y)+k(x,y)×2π

### 2.2. Motion Vector Estimation

The motion vectors rely on the 3D coordinate data obtained from a 3D measurement system. Firstly, we need to calculate the mask of the motion target.

(5) $$Q(X,Y,t)=\left\{\begin{array}{cc} 1 & \left(Z(X,Y)>a\right)\\ 0 & \left(otherwise\right) \end{array}\right.$$

where *a* represents the threshold for extracting the region of the moving target; the value of *a* is typically determined by averaging the fringe patterns and then selecting an appropriate threshold. Z(X,Y) represents the point cloud depth matrix at this moment. The corresponding mask can be extracted through an element-wise comparison with a predefined threshold.

Then, the 3D coordinates of the moving object at time *t* can be obtained as $Z^{\prime }(X,Y,t)=Z(X,Y,t)\cdot Q(X,Y,t)$. Based on this, the centroid information of the target $\left(\bar{X}(t),\bar{Y}(t),\bar{Z}(t)\right)$can be calculated as follows:

(6) $$\begin{array}{ccc} \bar{X}(t)=\frac{M_{x}}{M_{0}} & \bar{Y}(t)=\frac{M_{y}}{M_{0}} & \bar{Z}(t)=\frac{M_{z}}{M_{0}} \end{array}$$

The specific calculation of $M_{0}$, $M_{x}$, $M_{y}$ is as follows:

(7) $$\begin{array}{cc} M_{0}=\sum_{X,Y}Q(X,Y,t) & M_{z}=\sum_{X,Y}Z^{\prime }(X,Y,t)\\ M_{X}=\sum_{X,Y}Q(X,Y,t)\cdot X & M_{Y}=\sum_{X,Y}Q(X,Y,t)\cdot Y \end{array}$$

From the above equations, the centroids of the 3D point at different times can be estimated. Finally, the motion vectors (∇x,∇y,∇z) can be estimated through the movement of the centroids at different times.

### 2.3. Camera-Pixel Mismatch Correction

In 3D reconstruction using phase shifting, camera-pixel displacement and phase-shifting errors coexist in adjacent fringes. Correcting camera-pixel mismatches is crucial before phase retrieval. In the three-step phase-shifting algorithm, mismatches occur every three frames. The estimated motion vectors from step 2 can be used to correct these errors.

To solve the problem, it is necessary to find and adjust the pixel offsets for each fringe, so that the same pixel in all three images corresponds to the same point:

(8) $$\begin{array}{l} {u_{1}}^{c}=u^{c},{v_{1}}^{c}=v^{c}\\ {u_{2}}^{c}=u^{c}+{\nabla^{u}}_{12}(u^{c},v^{c}),{v_{2}}^{c}=v^{c}+{\nabla^{v}}_{12}(u^{c},v^{c})\\ {u_{3}}^{c}=u^{c}+{\nabla^{u}}_{13}(u^{c},v^{c}),{v_{3}}^{c}=v^{c}+{\nabla^{v}}_{13}(u^{c},v^{c}) \end{array}$$

In Equation (8), using the three-frequency, three-step phase-shifting method as an example, the three images shown represent the first three fringe images. In practice, pixel mismatch correction needs to be applied to all nine fringe images. $u_{1}^{c}$ and $v_{1}^{c}$ represent the horizontal and vertical coordinates of the first fringe image pixel, respectively. ${\nabla^{u}}_{12}(u^{c},v^{c})$ and ${\nabla^{v}}_{12}(u^{c},v^{c})$ denote the horizontal and vertical displacements of the second fringe image relative to the first. Similarly, ${\nabla^{u}}_{13}(u^{c},v^{c})$ and ${\nabla^{v}}_{13}(u^{c},v^{c})$ represent the horizontal and vertical displacements of the third fringe image relative to the first.

To determine the pixel offsets caused by object motion, we can start from the pinhole camera model as follows [20]:

(9) $$s^{z}\left[\begin{array}{l} u_{c}\\ v_{c}\\ 1 \end{array}\right]=A^{c}[R^{c},T^{c}]\left[\begin{array}{l} x_{w}\\ y_{w}\\ z_{w}\\ 1 \end{array}\right]=\left[\begin{array}{cccc} \begin{array}{l} m_{11}\\ m_{21}\\ m_{31} \end{array} & \begin{array}{l} m_{12}\\ m_{22}\\ m_{32} \end{array} & \begin{array}{l} m_{13}\\ m_{23}\\ m_{33} \end{array} & \begin{array}{l} m_{14}\\ m_{24}\\ m_{34} \end{array} \end{array}\right]\left[\begin{array}{l} x_{w}\\ y_{w}\\ z_{w}\\ 1 \end{array}\right]$$

This model represents the relationship between a given world coordinate point $\left(x_{w},y_{w},z_{w}\right)$ and its corresponding camera-pixel $\left(u^{c},v^{c}\right)$. Now, if a point moves from $\left(x_{w},y_{w},z_{w}\right)$ to $\left(x_{w}+\nabla x,y_{w}+\nabla y,z_{w}+\nabla z\right)$, the corresponding pixel will shift from $\left(u^{c},v^{c}\right)$ to $\left({\bar{u}}^{c},{\bar{v}}^{c}\right)$. The camera-pixel shift can be modeled using the following mathematical expressions:

$$\left\{\begin{array}{l} {\bar{u}}^{c}=u_{c}+\frac{\partial u(x_{w},y_{w},z_{w})}{\partial x_{w}}\nabla x+\frac{\partial u(x_{w},y_{w},z_{w})}{\partial y_{w}}\nabla y+\frac{\partial u(x_{w},y_{w},z_{w})}{\partial z_{w}}\nabla z\\ {\bar{v}}^{c}=v_{c}+\frac{\partial v(x_{w},y_{w},z_{w})}{\partial x_{w}}\nabla x+\frac{\partial v(x_{w},y_{w},z_{w})}{\partial y_{w}}\nabla y+\frac{\partial v(x_{w},y_{w},z_{w})}{\partial z_{w}}\nabla z\\ \frac{\partial u(x_{w},y_{w},z_{w})}{\partial x_{w}}=\frac{m_{11}(m_{31}x_{w}+m_{32}y_{w}+m_{33}z_{w}+m_{34})-m_{31}(m_{11}x_{w}+m_{12}y_{w}+m_{13}z_{w}+m_{14})}{(m_{31}x_{w}+m_{32}y_{w}+m_{33}z_{w}+m_{34}{)}^{2}}\\ \frac{\partial u(x_{w},y_{w},z_{w})}{\partial y_{w}}=\frac{m_{12}(m_{31}x_{w}+m_{32}y_{w}+m_{33}z_{w}+m_{34})-m_{32}(m_{11}x_{w}+m_{12}y_{w}+m_{13}z_{w}+m_{14})}{(m_{31}x_{w}+m_{32}y_{w}+m_{33}z_{w}+m_{34}{)}^{2}}\\ \frac{\partial u(x_{w},y_{w},z_{w})}{\partial z_{w}}=\frac{m_{13}(m_{31}x_{w}+m_{32}y_{w}+m_{33}z_{w}+m_{34})-m_{33}(m_{11}x_{w}+m_{12}y_{w}+m_{13}z_{w}+m_{14})}{(m_{31}x_{w}+m_{32}y_{w}+m_{33}z_{w}+m_{34}{)}^{2}}\\ \frac{\partial v(x_{w},y_{w},z_{w})}{\partial x_{w}}=\frac{m_{21}(m_{31}x_{w}+m_{32}y_{w}+m_{33}z_{w}+m_{34})-m_{31}(m_{21}x_{w}+m_{22}y_{w}+m_{23}z_{w}+m_{24})}{(m_{31}x+m_{32}y_{w}+m_{33}z_{w}+m_{34}{)}^{2}}\\ \frac{\partial v(x_{w},y_{w},z_{w})}{\partial y_{w}}=\frac{m_{22}(m_{31}x_{w}+m_{32}y_{w}+m_{33}z_{w}+m_{34})-m_{32}(m_{21}x_{w}+m_{22}y_{w}+m_{23}z_{w}+m_{24})}{(m_{31}x_{w}+m_{32}y_{w}+m_{33}z_{w}+m_{34}{)}^{2}}\\ \frac{\partial v(x_{w},y_{w},z_{w})}{\partial z_{w}}=\frac{m_{23}(m_{31}x_{w}+m_{32}y_{w}+m_{33}z_{w}+m_{34})-(m_{21}x_{w}+m_{22}y_{w}+m_{23}z_{w}+m_{24})}{(m_{31}x_{w}+m_{32}y_{w}+m_{33}z_{w}+m_{34}{)}^{2}} \end{array}\right.$$

After completing steps 1 to 3, the camera-pixel mismatch error is successfully eliminated, ensuring accurate alignment of the captured pixel data with the object’s actual geometry. This correction is crucial for minimizing one of the primary sources of distortion in dynamic 3D measurements. However, despite this improvement, residual motion-induced phase-shifting errors remain in the system. These errors, often manifested as periodic distortions in the reconstructed surface, are caused by discrepancies introduced during the phase-shifting process due to object motion. Such errors can significantly compromise the accuracy and reliability of the 3D reconstruction, particularly in scenarios involving complex or multidirectional motion.

To address these residual errors, step 4 focuses on applying advanced error compensation techniques. Specifically, the Hilbert transform is employed to shift the phase of the fringe patterns by π/2, enabling the identification and elimination of periodic phase errors. This step ensures that the reconstructed 3D surface is free from distortions caused by phase inconsistencies. Furthermore, the process includes iterative evaluation and refinement to ensure convergence and optimal correction. By systematically addressing these residual phase errors, step 4 plays a critical role in achieving high-accuracy 3D reconstructions, even under challenging dynamic conditions.

### 2.4. Phase-Shift Error Compensation Using the Hilbert Transform

After completing the previous steps, the camera-pixel mismatch problem is effectively alleviated, significantly improving the alignment between the captured pixel data and the object’s actual spatial configuration. However, residual errors persist, primarily originating from the projection subsystem. These errors are particularly critical in the context of phase-shifting algorithms, where precise phase shifts are essential for accurate 3D reconstruction. Object motion introduces deviations in the phase shift, rendering it imprecise and resulting in periodic phase errors. These errors occur because the relative motion between the object and the measurement system disrupts the synchronized relationship between projected fringe patterns and their captured counterparts. Such disruptions lead to inaccuracies in phase calculations, particularly in dynamic scenarios involving rapid or multidirectional motion. The periodic nature of these errors further complicates the reconstruction process, as they propagate across the reconstructed surface, creating distortions that undermine the fidelity of the 3D model.

In the phase-shifting algorithm, the phase shift $\delta_{n}$ is no longer precise, since object motion will cause error ε:

(10) $${\delta_{n}}^{\prime }=\delta_{n}+\varepsilon$$

The phase-shifting error, if not well suppressed, will result in a phase error [21]:

(11) $$\begin{array}{l} \nabla \phi =\phi (x,y{)}^{\prime }-\phi (x,y)\\ \approx \tan^{-1}[\frac{-\sqrt{3}\varepsilon \sin2\phi }{3+\sqrt{3}\varepsilon \cos\varepsilon 2\phi }]\\ \approx \tan^{-1}[\varepsilon \frac{-\sqrt{3}\sin2\phi }{3}]\\ \approx (-\frac{\sqrt{3}}{3}\varepsilon )\sin2\phi \end{array}$$

$\phi {\left(x,y\right)}^{\prime }$ is the phase error, and ϕ(x,y) is the true phase.

It can be observed that the phase error is approximately a sinusoidal function with a frequency twice that of the projected fringe. To eliminate these periodical phase error, we utilize the Hilbert transform to generate the complementary phase map, as follows:

(12) $$\phi^{H}(x,y)=\tan^{-1}\left[\frac{\sum_{n=1}^{N}I_{n}^{H}\cos{\delta^{\prime }}_{n}}{\sum_{n=1}^{N}I_{n}^{H}\sin{\delta^{\prime }}_{n}}\right]$$

where $I_{n}^{H}$ represents the fringes after the Hilbert transform $I_{n}^{H}=H[I_{n}]$.

By averaging the origin phase and the phase obtained through the Hilbert transform, the phase error can be eliminated as follows:

(13) $$\phi^{f}(x,y)=[\phi (x,y)+\phi^{H}(x,y)]/2$$

At this point, the phase values $\phi^{f}(x,y)$ obtained theoretically represent the phase error-corrected values. By putting these into Equation (2), the 3D reconstruction result corrected for motion error can be obtained.

Each iteration of steps 1 to 4 progressively refines the 3D reconstruction by systematically addressing errors introduced at different stages of the process. Meanwhile, the 3D centroids of the point clouds at different time points are calculated, and with each iteration, the centroids should progressively move closer to each other until a predefined minimum threshold is reached, at which point the iteration loop can be terminated. In each cycle, pixel alignment is improved, and phase-shifting errors are minimized, bringing the reconstructed model closer to the true spatial geometry of the object. This iterative approach ensures that residual errors are incrementally reduced, resulting in enhanced accuracy of the 3D reconstruction.

To monitor the progress of the iterations, convergence criteria are established, such as a predefined threshold for error reduction or stability in the reconstructed phase map across successive iterations. By evaluating these criteria at each step, the algorithm determines whether further iterations are necessary. This ensures computational efficiency by preventing unnecessary processing once the reconstruction has achieved optimal fidelity.

Additionally, this iterative refinement not only compensates for motion-induced errors, but also enhances robustness against external factors, such as noise and minor calibration inaccuracies. The process continues until the results stabilize, indicating that the reconstruction has reached its best achievable accuracy. The combination of iterative improvement and convergence monitoring provides a reliable framework for obtaining precise 3D measurements, even under challenging dynamic conditions.

## 3. Experiments and Discussion

To validate the effectiveness of the proposed method, we designed and implemented a phase-shifting profilometry (PSP) system equipped with a GVD PDC03 projector (800 × 1200 pixels) and an IDS UI-124XSE-M camera (1600 × 1200 pixels), both operating at a synchronized frame rate of 120 Hz. This high frame rate ensured that the system could capture dynamic scenes with minimal motion blur, providing a robust platform for testing the algorithm under various motion scenarios. The phase unwrapping process in the system employed a three-frequency phase-shifting algorithm, which is widely recognized for its ability to handle complex phase distributions and resolve ambiguity in phase retrieval. This algorithm allowed the system to accurately reconstruct the surface profile of objects by sequentially projecting three fringe patterns of varying frequencies onto the object surface. The reflected patterns were captured by the camera and analyzed to extract precise phase information. Additionally, to simulate realistic conditions and further evaluate the robustness of the method, the system was configured to measure objects undergoing controlled motion at different velocities and directions. This setup enabled us to assess the performance of the algorithm not only in static conditions, but also under dynamic environments, ensuring its applicability in real-world scenarios where motion-induced errors are prevalent. By combining advanced hardware and algorithmic techniques, the experimental setup provided a comprehensive framework for testing and validating the proposed motion error compensation method.

**Figure 3:**
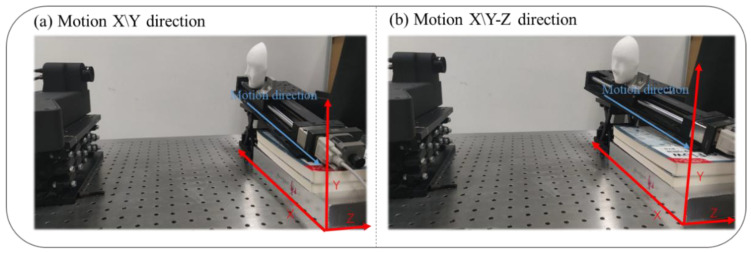
Experimental setup.

Figure 3 shows our experimental setup. To demonstrate the effectiveness of our algorithm when the measured object moves in different directions, we set up two motion scenarios: motion along the X/Y (X and Y) directions, as shown in Figure 3a, and motion along the X/Y-Z (X, Y, and Z) directions, as shown in Figure 3b.

### 3.1. Dynamic Reconstruction Accuracy

In the experiment, a standard ball was moved along the X/Y and Z axes.

**Figure 4:**
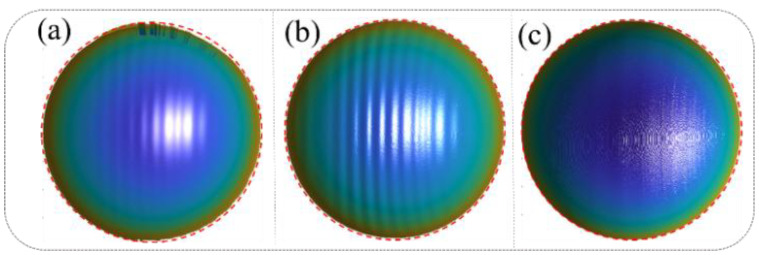
Standard ball random motion experiment results: (**a**) Three-dimensional reconstruction results of the standard ball’s random motion without any correction. (**b**) Three-dimensional reconstruction results of the standard ball’s random motion after correcting only the camera-pixel mismatch. (**c**) Three-dimensional reconstruction results of the standard ball’s random motion after simultaneously compensating for pixel mismatch and phase-shift errors.

Figure 4 presents a comparative analysis of the 3D reconstruction results under different error compensation conditions, demonstrating the progressive improvements achieved through the proposed method. Figure 4a illustrates the 3D reconstruction results obtained using the traditional phase-shifting algorithm without any error compensation. Significant edge distortions are evident in the reconstruction, particularly in areas with rapid transitions, indicating the detrimental effects of motion-induced errors on the accuracy of the measurement. Quantitatively, the mean error in this case is 0.142 mm, with a root mean square error (RMSE) of 0.453 mm, highlighting the limitations of the conventional algorithm in dynamic scenarios. Figure 4b presents the reconstruction results after applying correction for camera-pixel mismatch alone. While this step reduces edge distortions to some extent, it fails to fully address the motion-induced errors, as periodic sinusoidal phase errors persist across the surface of the reconstructed object. These residual errors underscore the need for a more comprehensive approach. The mean error in this case increases to 0.673 mm, with an RMSE of 0.863 mm, indicating that correcting only camera-pixel mismatches is insufficient for achieving high-accuracy reconstructions. Figure 4c showcases the results after simultaneously compensating for both camera-pixel mismatch and phase-shifting errors using the proposed method. The reconstruction demonstrates a marked improvement in accuracy, with edge distortions effectively eliminated and the sinusoidal phase errors significantly mitigated. This dual-correction approach achieves a mean error of just 0.0296 mm and an RMSE of 0.252 mm, representing a substantial enhancement compared to the previous cases. These results validate the effectiveness and robustness of the proposed algorithm in addressing motion-induced errors in dynamic 3D measurement scenarios. The comparison in Figure 4 highlights the progressive benefits of integrating camera-pixel mismatch correction with phase-shift error mitigation, emphasizing the importance of a comprehensive error compensation strategy for achieving precise and reliable 3D reconstructions in complex motion conditions. In this study, we built a more general-purpose 3D structured light imaging system, consisting of the GVD PDC03 projector (800 × 1200 pixels) and the IDS UI-124XSE-M camera (1600 × 1200 pixels), and compared the performance of our motion compensation algorithm with other algorithms on the same system. This was carried out to demonstrate that the compensation accuracy of our algorithm has a certain level of generalizability. Of course, different structured light imaging systems inherently have varying levels of precision. However, the 3D measurement system used in our experiments achieves an accuracy of better than 0.03 mm when measuring static objects. Additionally, with the application of our motion compensation algorithm, we are able to achieve similar high-precision measurements (within 0.03 mm) in dynamic scenes. Therefore, the algorithm’s accuracy is also influenced by the inherent measurement accuracy of the system, and it will improve as the original system’s accuracy improves.

### 3.2. Dynamic Reconstruction in Complex Scenarios

To evaluate the robustness of our proposed method under varying motion velocities, we conducted a series of 3D reconstruction experiments. These experiments involved scenarios where the mask moved at three distinct speeds, denoted as V1, V2, V3, along two different motion directions. These speeds were chosen to represent a range of motion dynamics, from relatively slow movements to faster, more challenging scenarios that test the limits of motion compensation techniques. The experimental setup allowed us to systematically analyze the performance of our method under diverse motion conditions and assess its ability to maintain reconstruction accuracy across varying velocities.

Furthermore, to highlight the superior generalizability and effectiveness of our proposed method compared to existing motion compensation approaches, we performed a comprehensive comparative analysis. This analysis included reconstruction results obtained using our algorithm, as well as those achieved through motion error compensation with the Hilbert transform, a commonly employed technique in the field. By comparing the outcomes, we aimed to demonstrate how our approach not only addresses camera-pixel mismatches, but also effectively mitigates phase-shifting errors, which are often inadequately handled by traditional methods.

The comparative analysis was conducted across various motion conditions, encompassing both unidirectional and multidirectional motion patterns, as well as scenarios with different velocity profiles. This allowed us to evaluate the adaptability of our method for handling complex and dynamic motion environments. The experimental results underscore the robustness of our approach, showing that it consistently outperforms conventional methods in terms of reconstruction accuracy and error mitigation, regardless of the motion conditions.

**Figure 5:**
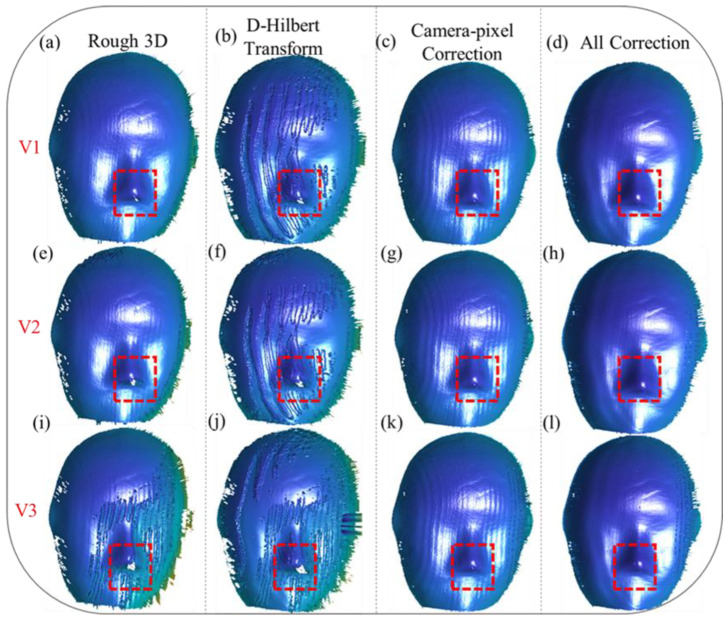
Three-dimensional reconstruction of the mask moving along the X/Y-Z directions at different velocities (V1, V2, V3). (**a**,**e**,**i**) depict the rough 3D reconstruction results of the mask’s point clouds. (**b**,**f**,**j**) show the reconstruction results obtained by directly applying the Hilbert transform for error correction. (**c**,**g**,**k**) present the reconstruction results after correcting only the camera-pixel mismatch. (**d**,**h**,**l**) demonstrate the reconstruction results after simultaneously compensating for both camera-pixel mismatch and phase-shifting errors.

As illustrated in Figure 5a,e,i, motion along the X and Y directions (X/Y) introduces significant errors in the initial 3D reconstructions. These errors are particularly pronounced as the velocity increases, leading to the emergence of periodic distortions on the reconstructed mask surface. These distortions disrupt the smoothness and continuity of the reconstructed surface, affecting the overall accuracy and reliability of the measurement. In addition to the periodic errors, the edge regions, as highlighted by the red boxes, suffer from severe issues of missing data caused by pixel shifting. These issues become increasingly problematic at higher velocities, as the rapid motion exacerbates the misalignment between the captured data and the actual object geometry.

Applying the Hilbert transform alone, as shown in Figure 5b,f,j, demonstrates its limitations in resolving these issues. While it can partially reduce periodic distortions, it proves inadequate for addressing the missing data problems in the edge regions, particularly in areas where motion effects are more pronounced. This underscores the necessity of addressing camera-pixel mismatches as a fundamental step in error compensation. Without correcting this mismatch, the reconstruction remains prone to inaccuracies, limiting its applicability in high-precision scenarios.

When the camera-pixel mismatch is corrected, as depicted in Figure 5c,g,k, significant improvements in reconstruction accuracy can be observed. Pixel misalignment-induced errors are effectively eliminated, and missing reconstruction details in the edge regions, particularly within the red-boxed areas, are successfully restored. The corrected reconstruction provides a more accurate representation of the object’s geometry, even under motion conditions. However, despite these advancements, periodic phase errors persist on the mask surface, indicating that addressing pixel mismatches alone is insufficient for achieving high-accuracy results.

Finally, as demonstrated in Figure 5d,h,l, our proposed method provides a comprehensive solution by simultaneously addressing both camera-pixel mismatch and phase-shifting errors. This dual-compensation approach resolves the missing data issues in the edge regions, completely restoring the lost details, and significantly mitigates the periodic distortions across all three velocities. The integration of these compensation steps results in smoother and more accurate 3D reconstructions, free from the limitations observed in previous approaches. These results highlight the robustness and effectiveness of our method for achieving high-fidelity reconstructions, even under challenging dynamic conditions. This comprehensive correction process not only ensures accurate measurements in complex motion scenarios, but also showcases the potential of our method for broader applications in dynamic 3D measurement tasks.

We also tested a motion scenario with the object moving along the X/Y-Z (X, Y, and Z) directions, and the results are illustrated in the following figure.

**Figure 6:**
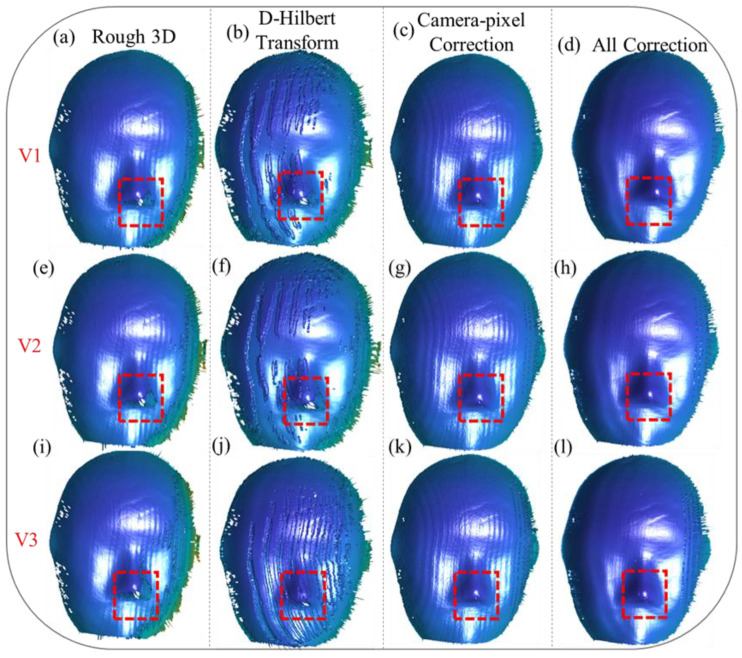
Three-dimensional reconstruction of the mask moving along the X/Y-Z directions at different velocities (V1, V2, V3). (**a**,**e**,**i**) depict the rough 3D reconstruction results of the mask’s point clouds. (**b**,**f**,**j**) show the reconstruction results obtained by directly applying the Hilbert transform for error correction. (**c**,**g**,**k**) present the reconstruction results after correcting only the camera-pixel mismatch. (**d**,**h**,**l**) demonstrate the reconstruction results after simultaneously compensating for both camera-pixel mismatch and phase-shifting errors.

Figure 6a,e,i depict the initial 3D reconstruction results at different velocities, namely V1, V2, V3, showcasing the effects of motion on reconstruction accuracy. For motion along the X/Y-Z directions, the reconstructions reveal even more pronounced periodic errors compared to those along the X/Y directions, particularly as velocity increases. These periodic distortions are most evident on the mask surface, where the structured patterns exhibit noticeable deviations. Additionally, in the edge regions, as highlighted by the red boxes, the inaccuracies in the reconstruction become increasingly severe as velocity rises, leading to substantial data loss and misalignment issues.

Applying the Hilbert transform alone, as shown in Figure 6b,f,j, demonstrates its limited ability to address these errors. While this approach reduces some periodic distortions, it fails to eliminate them completely, especially in the edge regions, where pixel misalignment causes significant data loss. This highlights the critical need to address camera-pixel mismatches as a fundamental step in the error correction process.

When the camera-pixel mismatch is corrected, as shown in Figure 6c,g,k, there is a noticeable improvement in the reconstruction. Errors caused by pixel misalignment are effectively eliminated, and missing reconstruction details in the edge regions, particularly within the red-boxed areas, are successfully restored. Despite these advancements, periodic errors on the mask surface remain unresolved, indicating the limitations of addressing pixel mismatches alone.

Ultimately, our proposed method, as depicted in Figure 6d,h,l, provides a comprehensive correction that simultaneously compensates for camera-pixel mismatch and phase-shift errors. This dual-correction approach fully resolves inaccuracies in the edge regions, completely restoring the lost data, while significantly suppressing periodic errors across all velocities. The results clearly demonstrate the robustness and efficacy of the proposed method for handling complex motion scenarios, achieving precise and high-quality 3D reconstructions even under challenging conditions. These findings validate the capability of the proposed algorithm to enhance reconstruction accuracy, making it a valuable solution for dynamic 3D measurement tasks.

**Figure 7:**
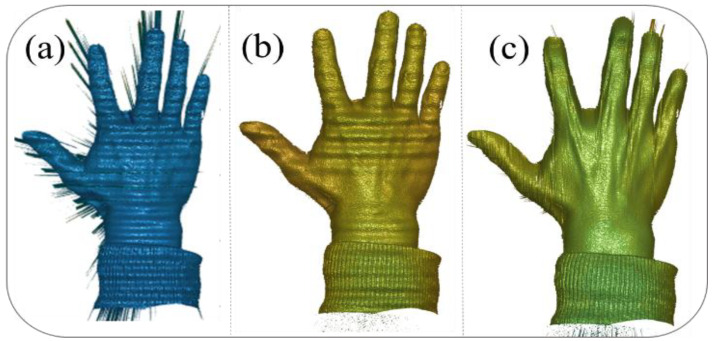
Moving hand reconstruction results: (**a**) Conventional phase-shifting algorithm reconstruction results without any correction. (**b**) Reconstruction results after correcting only the camera-pixel mismatch. (**c**) Reconstruction results after simultaneously compensating for pixel mismatch and phase-shift errors.

To further verify that our motion compensation method is not only effective for simple geometric shapes, but also applicable to complex objects, we selected a new reconstruction target for experimentation:

Figure 7 illustrates the reconstruction results for a moving hand under different correction strategies, highlighting the effectiveness of the proposed method. In Figure 7a, the reconstruction results using the conventional phase-shifting algorithm without any corrections exhibit significant distortions and inaccuracies, due to the presence of both camera-pixel mismatch and phase-shifting errors, particularly in regions of rapid motion. Figure 7b shows the results after correcting only the camera-pixel mismatch, where the pixel misalignment-induced errors are eliminated, leading to a clearer reconstruction. However, periodic phase errors remain visible, particularly on the surface of the hand, causing noticeable distortions. Finally, Figure 7c presents the reconstruction results after simultaneously compensating for both pixel mismatch and phase-shifting errors, demonstrating a significant improvement in accuracy. The hand’s surface is reconstructed with minimal distortions, and the previously prominent errors are effectively mitigated, showcasing the robustness and precision of the proposed method for handling dynamic scenes.

## 4. Conclusions

This paper presents a motion error compensation method for dynamic 3D measurement, with experimental results showing a reconstruction accuracy of 0.03 mm. This method effectively addresses motion-induced errors, which are common in high-precision applications. Achieving this level of accuracy is important for the precise measurement and modeling of dynamic objects, particularly in fields such as robotics, medical imaging, and industrial inspection. Unlike traditional algorithms that often rely on strict assumptions about object motion and are limited to specific scenarios, this approach offers a flexible and adaptable solution. By simultaneously addressing camera-pixel mismatches and phase-shifting errors in 3D space, it enables reliable 3D reconstructions, even under complex and dynamic motion conditions. A key advantage of this method is its ability to compensate for motion-induced errors without requiring high-speed camera systems. Traditional approaches typically address motion errors by increasing the acquisition speed of fringe images, which often results in higher hardware costs. In contrast, this method uses mismatches in adjacent fringe images to correct errors, even with slower camera setups. This makes the method more cost-effective and suitable for a wider range of applications, especially in scenarios where hardware upgrades may not be possible. The core of the algorithm lies in its dual-error compensation strategy. It first addresses camera-pixel mismatches, a significant source of reconstruction inaccuracies in dynamic scenes. Additionally, the method compensates for phase-shifting errors, which are particularly problematic in complex or multidirectional motion scenarios. Using advanced mathematical tools such as the Hilbert transform, the algorithm reduces periodic phase errors, helping to restore the smoothness and integrity of the reconstructed surface. Experimental validation demonstrates the effectiveness of this approach in a range of motion directions and velocities. The results show notable improvements in reconstruction accuracy compared to conventional methods, particularly in situations where traditional approaches face challenges. For instance, when compared to traditional phase-shifting methods or those that only correct phase errors, the proposed method shows reductions in point cloud gaps, order errors, and edge distortions. These improvements are important because they enhance the fidelity of the reconstructed model, ensuring that the object’s geometry is represented more accurately, and addressing issues like incomplete point clouds or distortion that can negatively affect measurement accuracy. The ability of the algorithm to handle both low-speed and high-speed motions with consistent precision highlights its robustness and adaptability. Low-speed motion, in this context, is defined as pixel-level errors caused by object movement within a range of one pixel, where reconstruction accuracy remains high. High-speed motion, however, refers to scenarios where the pixel mismatches exceed one pixel, leading to significant distortion in the reconstructed model if not corrected. Therefore, we defined three different speeds, V1–V3 (0.03 mm/s, 0.7 mm/s, and 0.1 mm/s), which correspond to the low and high speeds discussed in the paper. This method compensates for such errors, ensuring reliable reconstructions even in high-speed dynamic scenarios. By aligning pixel data with the actual geometry of the object, the method ensures that the captured data reflect the spatial configuration of the object more accurately. Additionally, the iterative optimization process helps the algorithm converge to an optimal solution, stopping once the difference between successive iterations is below a predefined threshold, ensuring stability and accuracy in the results. Future work could involve integrating more advanced motion vector analysis techniques to handle even more complex motion scenarios, such as rotational and translational movements of the target object. These enhancements would further extend the method’s applicability, making it more suitable for highly dynamic environments where objects undergo unpredictable or multi-axis motion. Such improvements could expand the potential of the method in motion error compensation technologies.

In summary, this motion error compensation method offers a promising approach for dynamic 3D measurement, addressing many limitations of traditional algorithms. It combines broad applicability with a cost-effective design, making it suitable for fields such as robotics, industrial inspection, medical imaging, and virtual reality. However, it is important to note that this method has certain limitations. It assumes that the object moves at a constant speed along a straight line, and the use of centroids in motion compensation limits its applicability to rigid body motion. As a result, the method may not be effective for dynamic 3D measurements of non-rigid body movements. Additionally, when the object at the edge of the field of view (FOV) moves too quickly, it may exceed the measurement range, causing certain points to become unmeasurable at different time instances. In such cases, our method assumes that the object remains within the FOV, and we have not addressed a scenario where the object moves too quickly to be captured. Indeed, in these situations, the motion compensation algorithm would fail. Despite these constraints, this method provides valuable insights and support for advancing the accuracy and efficiency of dynamic 3D measurement systems.

## Data Availability

Data available on request from the authors.
